# Contribution of compositional changes in the workforce to sickness absence trends in Finland: Elli Hartikainen

**DOI:** 10.1093/eurpub/ckac129.094

**Published:** 2022-10-25

**Authors:** T Leinonen, E Hartikainen, L Salonen, E Viikari-Juntura, S Solovieva

**Affiliations:** Finnish Institution of Occupational Health, Helsinki, Finland

## Abstract

**Background:**

Information on factors driving work disability trends helps to evaluate the potential of interventions to improve the health and work ability of the workforce. We assessed whether the long-term decrease in sickness absences in Finland is explained by observed and unobserved compositional changes in the workforce.

**Methods:**

Utilising register-based panel data on Finnish private and public sector employees aged 30-62, we examined the annual onset of compensated full sickness absence (granted after 10 working days) in the period 2005-2016. We applied random effects models adjusting for changes in observed sociodemographic factors of the study population. We also applied fixed effects models, with corrections of the estimates for cohort ageing, to additionally account for unobserved time-invariant characteristics of the study population over the years.

**Results:**

Of the observed factors, increases in educational level partly explained the decreasing trend in sickness absences, and more so among women than men and among private than public sector employees. Changes in occupational class and industrial sector played little role in the public sector and only slightly further explained the sickness absence trend in the private sector. The decreasing trend in sickness absences appeared to be largely explained by unobserved time-invariant individual characteristics.

**Conclusions:**

The decrease in sickness absences appeared to be more strongly influenced by compositional changes in factors that are established before fully entering the labour market - such as educational level as well as unmeasured individual characteristics that remain unchanged after childhood and early adulthood - than in the work environment or other factors contributing at working age. Attempts to improve the health and work ability of the workforce should not only rely on interventions directed at the working age population, but also on those carried out early during the life course.

**Key messages:**

• Compositional changes in the workforce should be taken into account when assessing sickness absence trends.

• Interventions aiming to improve the health and work ability of the workforce should be implemented already early in the life course and not only in working age.

